# Adverse events of intestinal microbiota transplantation in randomized controlled trials: a systematic review and meta-analysis

**DOI:** 10.1186/s13099-022-00491-3

**Published:** 2022-05-26

**Authors:** Chong Chen, Liyu Chen, Dayong Sun, Cailan Li, Shiheng Xi, Shihua Ding, Rongrong Luo, Yan Geng, Yang Bai

**Affiliations:** 1 Department of Gastroenterology, The First Affiliated Hospital of Shenzhen University, Shenzhen Second People’s Hospital, Shenzhen, 518037 China; 2 Department of Gastroenterology, 923Th Hospital of PLA Joint Logistics Support Force, Nanning, 530021 China; 3 Guangdong Provincial Key Laboratory of Gastroenterology, Department of Gastroenterology, Nanfang Hospital, Southern Medical University, Guangzhou, 510515 China

**Keywords:** Intestinal microbiota transplantation, Adverse events, Meta-analysis, Randomized controlled trials

## Abstract

**Background:**

Intestinal microbiota transplantation (IMT) has been recognized as an effective treatment for recurrent *Clostridium difficile* infection (rCDI) and a novel treatment option for other diseases. However, the safety of IMT in patients has not been established.

**Aims:**

This systematic review and meta-analysis was conducted to assess the safety of IMT.

**Methods:**

We systematically reviewed all randomized controlled trials (RCTs) of IMT studies published up to 28 February 2021 using databases including PubMed, EMBASE and the Cochrane Library. Studies were excluded if they did not report adverse events (AEs). Two authors independently extracted the data. The relative risk (RR) of serious adverse events (SAEs) and common adverse events (CAEs) were estimated separately, as were predefined subgroups. Publication bias was evaluated by a funnel plot and Egger’s regression test.

**Results:**

Among 978 reports, 99 full‐text articles were screened, and 20 articles were included for meta-analysis, involving 1132 patients (603 in the IMT group and 529 in the control group). We found no significant difference in the incidence of SAEs between the IMT group and the control group (RR = 1.36, 95% CI 0.56–3.31, *P* = 0.50). Of these 20 studies, 7 described the number of patients with CAEs, involving 360 patients (195 in the IMT group and 166 in the control group). An analysis of the eight studies revealed that the incidence of CAEs was also not significantly increased in the IMT group compared with the control group (RR = 1.06, 95% CI  0.91–1.23, *P* = 0.43). Subgroup analysis showed that the incidence of CAEs was significantly different between subgroups of delivery methods (*P*_(CAE)_ = 0.04), and the incidence of IMT-related SAEs and CAEs was not significantly different in the other predefined subgroups.

**Conclusion:**

Currently, IMT is widely used in many diseases, but its associated AEs should not be ignored. To improve the safety of IMT, patients' conditions should be fully evaluated before IMT, appropriate transplantation methods should be selected, each operative step of faecal bacteria transplantation should be strictly controlled, AE management mechanisms should be improved, and a close follow-up system should be established.

## Introduction

Gut microbiome dysbiosis is believed to be associated with numerous human diseases [1]. Thus, an increasing number of drugs and therapeutic methods targeting intestinal microecology, such as intestinal microbiota transplantation (IMT), have appeared in recent years [2]. IMT is used to rebuild the microbiota of the gastrointestinal tract by taking faeces from a strictly screened healthy donor and transplanting it into the gastrointestinal tract of the recipients through various delivery routes [3].

Currently, encouraged by the great success of IMT in the treatment of recurrent *Clostridium difficile* infection (rCDI) [4–6], researchers and clinicians have begun to explore the potential of IMT for the treatment of other diseases, including chronic constipation [7, 8], diarrhoea [9, 10], irritable bowel syndrome (IBS) [11–14], inflammatory bowel disease (IBD) [15–17], metabolic syndrome [18–20], obesity [21–23], immune system diseases [24, 25] and neurological/psychiatric system diseases (autism, anxiety, depression, epilepsy and Parkinson’s disease) [26–29]. Although the efficacy of the current treatment in other diseases is encouraging, few studies have focused on its potential adverse events (AEs), and some randomized controlled trials (RCTs) have not paid sufficient attention to the observation of AEs.

It is important to understand the potential risks of a new technology so that patients can be properly consulted and adequately evaluated before treatment. In addition, the summary and analysis of AEs of this treatment technique can improve the treatment protocols and make the treatment procedure more standardized. With the popularization and application of IMT technology, increasing AEs related to IMT have been reported [30, 31]. To further investigate the AEs of IMT in the treatment of diseases and to attract the attention of researchers and clinicians to the AEs related to IMT, a systematic review and meta-analysis of AEs in current reliable RCTs is necessary.

## Methods

This systematic review and meta-analysis was carried out in accordance with the Preferred Reporting Items for Systematic Reviews and Meta-analyses (PRISMA) guidelines [32].

### Data sources and search strategy

**Fig. 1:**
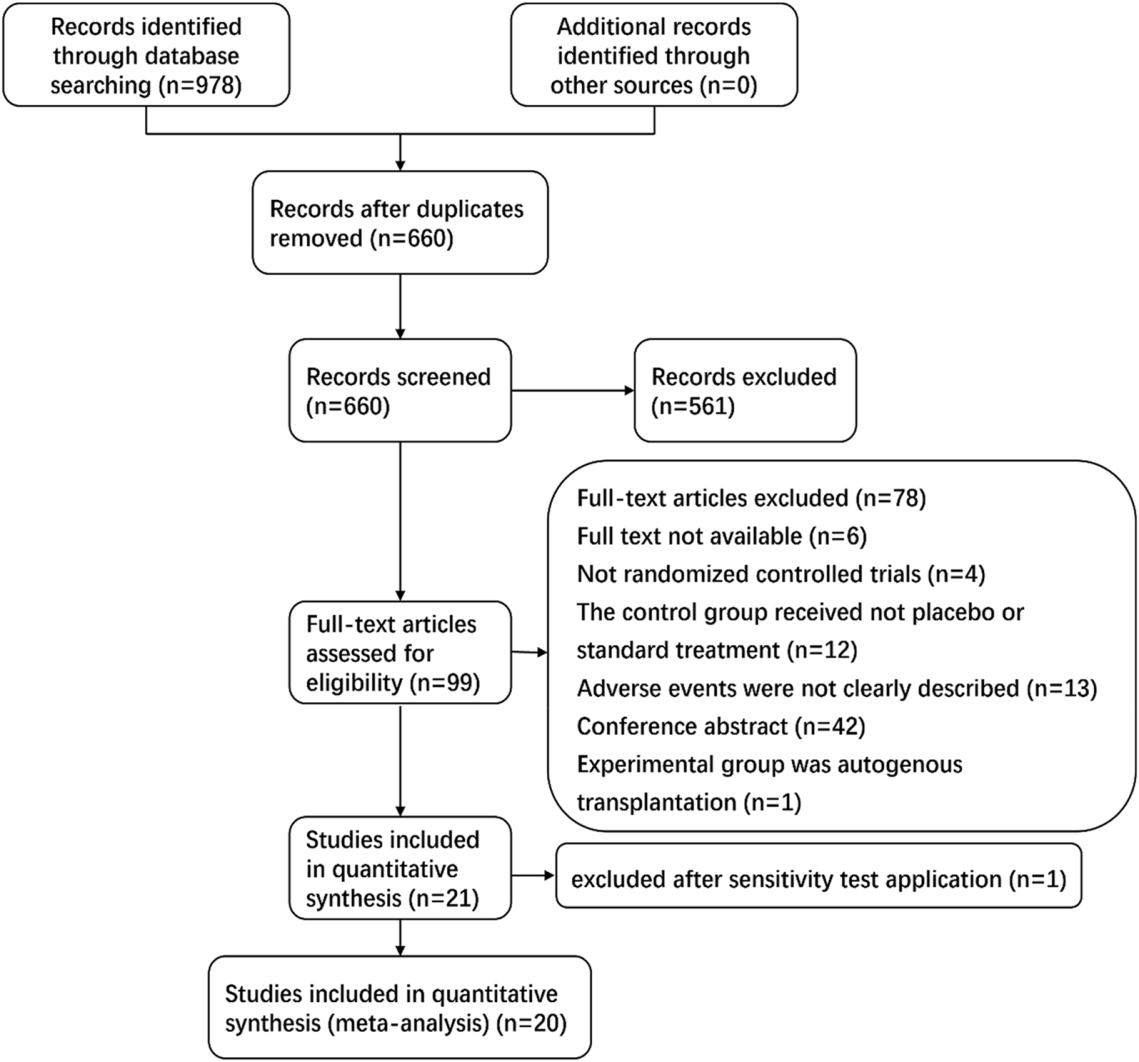
Flow diagram: meta-analysis of AEs of IMT in randomized controlled trials

Two independent reviewers (Chong Chen and Liyu Chen) searched electronic databases, including PubMed, EMBASE and the Cochrane Library. All databases were searched up to 28 February 2021. The search strategy was not limited by language. Abstract data were excluded, and only completed studies that underwent the full, rigorous peer-review process were included. The key terms were searched for in both free texts and medical subject headings (MeSH). The search terms used for fecal microbiota transplantation were as follows: “faecal” or “fecal” or “feces” or “faeces” or “stool” or “microbiota” or “microflora” or “fecal flora” or “faecal flora,” and “transplant*” or “transfusion” or “implant*” or “instillation” or “donor*” or “enema” or “reconstitution or infusion*” or “transfer*” or “FMT” or “bacteriotherapy.” The results were combined with “adverse”. A high sensitivity filter was used to limit studies to RCTs and humans. Figure 1 shows the flow diagram of study selection.

### Inclusion and exclusion criteria

Two independent authors (Chon Chen and Shiheng Xi) reviewed the title and abstract search with inclusion decisions for each article made independently based on the eligibility criteria. Inclusion criteria were as follows: (1) randomized controlled design; (2) studies conducted in humans rather than animals; (3) faecal transplantation offered in the interventional arm; and (4) control group treatment consisting of only the standard treatment, IMT excipient (no microbiota) or an autologous IMT. (Patients receiving IMT through different modalities were all permitted, as were studies that used either single- or pooled-donor IMT.) (5) AEs described in detail in the studies. Exclusion criteria were as follows: (1) articles that did not satisfy the inclusion criteria; (2) studies for which the full-text publication was not available; (3) conference abstracts; and (4) studies in which the experimental group received autologous transplantation.

### Study quality assessment

The quality of the studies was assessed independently by two authors (Yan Geng and Dayong Sun) according to the Cochrane Handbook for Systematic Review of Interventions [6]. When the two authors could not reach an agreement on an assessment, the third reviewer (Yang Bai) rendered the final decision. The contents of the assessment included the following seven points: (1) Random sequence generation or not or unclear? (2) Allocation concealment or not or unclear? (3) Blindness of participants and personnel or not or unclear? (4) Blindness of outcome assessment or not or unclear? (5) Incomplete outcome data or not or unclear? (6) Selective reporting or not or unclear? (7) Other bias. The GRADE system was used to estimate the overall quality of the evidence, and the integrity of the above seven points was regarded as the assessment standard. If the above bias did not exist, this was defined as low risk; the existence of the above bias was identified as high risk. If no reference was mentioned in the article, this was defined as unclear.

### Outcome of interest

This study focused on serious adverse events (SAEs) and common adverse events (CAEs) definitely/probably/possibly related to IMT. If the articles did not clearly describe whether the AEs were related to IMT, two authors (Chong Chen and Liyu Chen) discussed the studies together to decide whether to include them. If there was a disagreement, the third author (Yang Bai) rendered the decision. The severity of AE was categorized as follows according to the description of the articles or the Common Terminology Criteria for Adverse Events (CTCAE) v5.0: mild (grade 1), moderate (grade 2), severe (grade 3), life-threatening (grade 4) and death (grade 5) [31]; here, we considered grades 1 and 2 to indicate CAEs and grades 3, 4 and 5 to indicate SAEs.

### Data collection

Two authors (Shihua Ding and Cailan Li) independently extracted information from the included articles. The following data were collected for each study: (1) basic characteristics of the included articles: country of origin, study design, disease, route of delivery, follow‐up, donor source, antibiotics use before IMT, bowel preparation and the use of proton pump inhibitor (PPI) before IMT, stool sample dose, faecal status and frequency of IMT; (2) characteristics of the participants in the RCTs: sample size, sex ratio, mean age, the number of patients with SAEs and CAEs in the IMT and control groups, and the number of CAEs in the IMT and control groups; and (3) details regarding the SAEs.

### Statistical analysis

The relative risk (RR) ratio and 95% confidence interval were obtained by comparing the incidence of SAEs and CAEs in the IMT group to that in the control group. We tested for heterogeneity using the chi-square test and the *I*^2^ test. The chi-square test suggested heterogeneity between studies when the *P value* was less than 0.1. The *I*^2^ test describes the percentage of variability in effect estimates that is due to heterogeneity rather than chance, wherein an *I*^2^ test greater than 50% suggests substantial heterogeneity. When there was heterogeneity, a random effects model was used, and sensitivity analysis was needed. Otherwise, the fixed effect model was used. Subgroup analysis was applied to explore the potential relationship between IMT characteristics and AEs. All of the above statistical analyses were conducted in Review Manager 5.3 (RevMan; the Nordic Cochrane Centre). A funnel plot was applied to evaluate publication bias, along with Egger’s test, for which a p value less than 0.05 indicated potential publication bias.

## Results

### Search results and study characteristics

The search strategy identified 978 citations, of which 318 were duplicates. After title and abstract screening, the full texts of 99 relevant citations were selected for a thorough analysis. Thereafter, 78 citations were excluded for the following reasons: (1) the full text of 6 citations was not available; (2) 4 citations were not RCTs; (3) the control group in 12 studies did not receive placebo or standard treatment; (4) AEs of 13 citations were not clearly described; (5) 42 were conference abstracts; and (6) the experimental group was autogenous IMT in 1 citation, resulting in a total of 21 studies for the qualitative synthesis. Then, after a sensitivity analysis, we removed a study that was considered the main source of heterogeneity. Finally, 20 articles [12–14, 23, 24, 33–36, 38–48] were included for meta-analysis (Fig. 1), involving 1132 patients: 603 in the IMT group and 529 in the control group. The details and characteristics of the included studies are presented in Table 1. All of these studies were RCTs from published from 2015 to 2020, five of which were open-label RCTs, one of which was a single-blind RCT and fifteen of which were double-blind RCTs.

**Table 1:** Basic characteristics of the included articles

References	Country	Study design	Disease	Route of delivery	Follow up	Donor source	Antibiotics use before IMT	Preparation	Stool sample dose	Fresh or frozen	Frequency of IMT
Cammarota et al. [33]	Italy	Open-label RCT	rCDI	Colonoscopy	10 weeks	Healthy volunteers related or unrelated	Vancomycin	4 L macrogol	50 ml solution	Fresh	Fecal infusion every 3 days
Moayyedi et al. [34]	Canada	Double-blind RCT	UC	Enema	12 months	Healthy volunteers	NR	NR	50 g	Fresh	once per week for 6 weeks
Rossen et al. [35]	Netherlands	Double-blind RCT	UC	Nasoduodenal tube	12 weeks	Healthy partners, relatives or volunteers	NR	2 L macrogol	NR	Fresh	× 2
Kelly et al. [36]	USA	Double-blind RCT	rCDI	Colonoscopy	6 months	Healthy volunteers	Vancomycin	Sodium sulfate, potassium sulfate, magnesium sulfate	64 g	Fresh	× 1
Bajaj et al. [37]	USA	Open-label RCT	Hepatic encephalopathy	Enema	150 days	Healthy volunteers	Metronidazole, ciprofloxacin, amoxicillin	NR	90 ml	Frozen	× 1
Hota et al. [38]	Canada	Open-label RCT	rCDI	Enema	120 days	Healthy volunteers	Vancomycin	NR	50 g	Fresh	× 1
Paramsothy et al. [39]	Australia	Double-blind RCT	UC	Enema	8 weeks	Healthy volunteers	NR	NR	37.5 g	Frozen	× 5 per week for 8 weeks
Tian et al. [40]	China	Single-blind RCT	Constipation	Nasointestinal tube	12 weeks	Healthy volunteers	NR	NR	100 g	Frozen	many times
Halkjær et al. [12]	Denmark	Double-blind RCT	IBS	Capsules	6 months	Healthy volunteers	NR	NR	50 g	Frozen	25 capsules per day for 12 days
Johnsen et al. [13]	Norway	Double-blind RCT	IBS	Colonoscopy	12 months	Healthy volunteers	No antibiotics were given	Sodium picosulphate plus magnesium citrate	50–80 g	Fresh	× 1
Aroniadis et al. [14]	USA	Double-blind RCT	IBS-D	Capsules	24 weeks	Healthy volunteers	NR	PPI	9.5 g	Frozen	×3
Costello et al. [41]	Australia	Double-blind RCT	UC	Colonoscopy	12 months	Healthy volunteers	NR	Polyethylene glycol and loperamide	100 g	Frozen	×3
Huttner et al. [42]	France	Open-label RCT	Multidrug-resistant Enterobacteriaceae	Capsules or nasogastric	30 weeks	Healthy volunteers	Colistin and neomycin	PPI	40 g	Frozen	×2
Hvas et al. [43]	Denmark	Open-label RCT	rCDI	Colonoscopy or nasojejunal tube	8 weeks	Healthy volunteers	Vancomycin	Standard lavage	50 g	Frozen	×1
Sood et al. [44]	India	Double-blind RCT	UC	Colonoscopy	48 weeks	Healthy volunteers	NR	Polyethylene glycol	100 g	Frozen	×1 every 8 weeks for 48 weeks
Allegretti, et al. [23]	USA	Double-blind RCT	Obese	Capsules	12 weeks	Healthy lean donors	No antibiotics	PPI;No bowel preparation	22.5 g, 9 g, 9 g (week0, 4, 8)	Frozen	×3
Bajaj et al. [37]	USA	Double-blind RCT	Alcohol use disorder	Enema	6 months	Healthy volunteers	NR	NR	27 g	NR	×1
El-Salhy et al. [46]	Norway	Double-blind RCT	IBS	Gastroscope	3 months	Healthy volunteers	NR	NR	30 g, 60 g	Frozen	×1
Fretheim et al. [24]	Norway	Double-blind RCT	Systemic sclerosis	Gastroscope	16 weeks	Healthy volunteers	NR	NR	NR	Frozen	×1
Lahtinen et al. [47]	Finland	Double-blind RCT	IBS	Colonoscopy	52 weeks	Healthy volunteers	NR	NR	NR	Frozen	×1
Yu et al. [48]	USA	Double-blind RCT	Metabolism in obesity	Capsules	12 weeks	Healthy lean donors	NR	NR	Total 105 capsules	Frozen	×2 first week×1/week for 5 weeks

*IMT* intestinal microbiota transplantation, *RCT* randomized controlled trial, *rCDI* recurrent Clostridioides difficile infection, *NR* not reported, *IBS* Inflammatory Bowel Disease, *UC* Ulcerative colitis, *PPI* proton pump inhibitor

### Bias risk assessment of the included RCTs

**Fig. 2:**
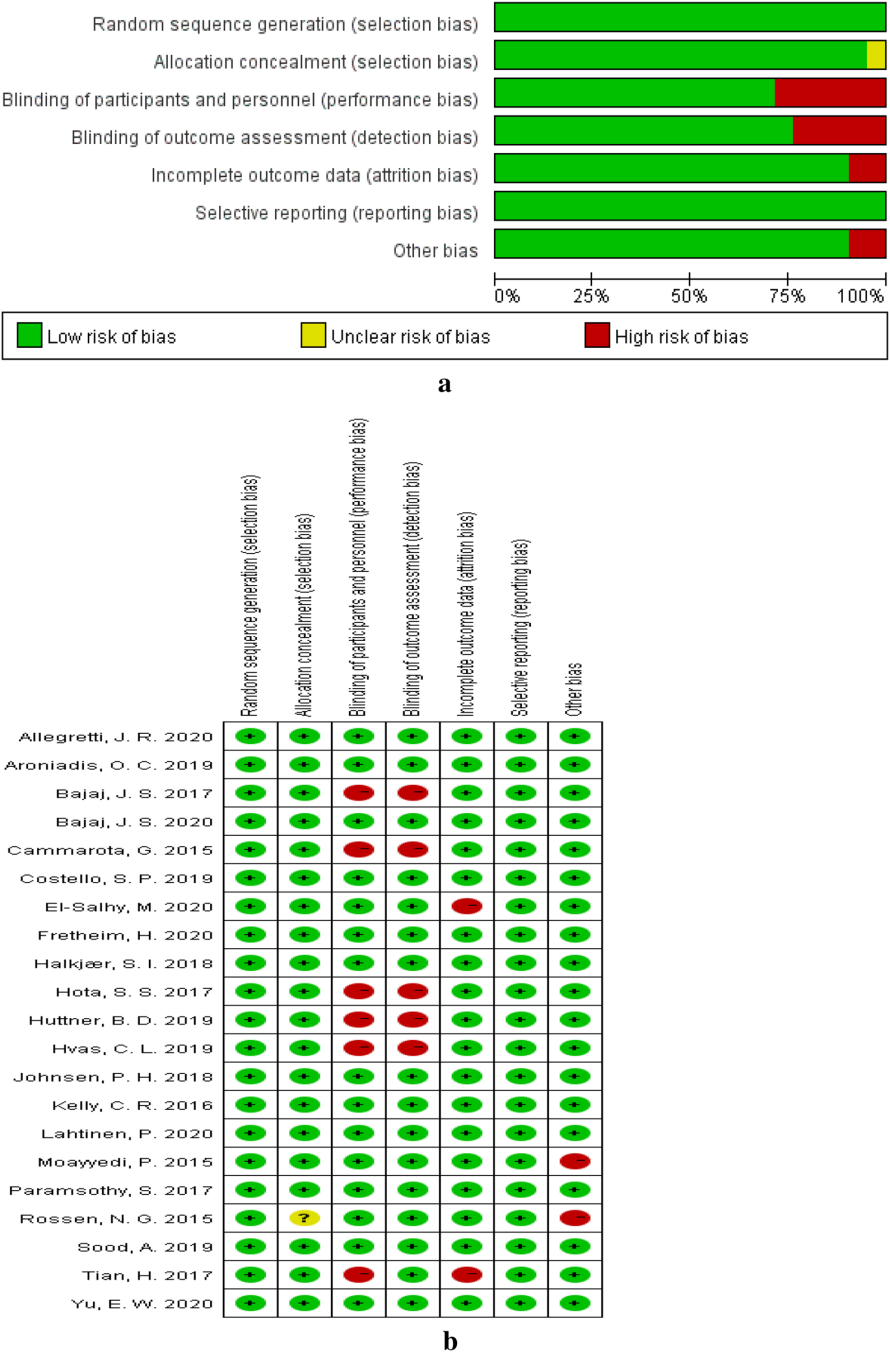
**a** Risk of bias graph. **b** Risk of bias summary. “ + ” indicates study meets criteria. “?” indicates unclear if study meets criteria. “-” indicates study not meets criteria

According to the guidelines of the Cochrane intervention system evaluation manual, 13 studies had a low risk of bias [12–14, 23, 24, 36, 39, 41, 44, 45, 47, 48]. Five of the studies were considered high risk because the blind method was not used [33, 37, 38, 42, 43]. One trial was considered high risk because the method was blinded only to the investigator [40], not to the subjects, and the reason for the loss of follow-up was not stated. Another study also did not state the reason for loss to follow-up [46]. In one study [34], the Data Monitoring and Safety Committee advised that the trial should be discontinued, as the treatments were futile. In addition, another study was terminated early due to an interim futility analysis and considered to have an unclear risk of bias because the allocation concealment was not explained [35]. Details of the risk-of-bias assessment by domain for each trial are shown in Fig. 2.

### Meta-analysis of the AEs of IMT

The characteristics of the participants and the number of patients with SAEs and CAEs are listed in Table 2. A total of 1132 patients were included in this study: 603 in the IMT group and 529 in the control group. The male-to-female ratio was 248:345 in the IMT group and 240:279 in the control group. The mean age of participants in all studies ranged from 33 to 75.7 years.

**Table 2:** The characteristics of the participants in RCTs

Diseases	References	Sample size		Sex(M:F)		Mean age		SAEs (patients)		CAEs (patients)		CAEs (events)	
		IMT	CON	IMT	CON	IMT	CON	IMT	CON	IMT	CON	IMT	CON
rCDI	Cammarota et al. [33]	20	19	08:12	08:11	71	75	0	0	NR	NR	43	0
	Kelly et al. [36]	22	24	04:18	05:19	48	55	0	0	NR	NR	NR	NR
	Hota et al. [38]	16	12	05:11	04:08	75.7	69.6	0	0	NR	NR	88	95
	Hvas et al. [43]	24	40	04:20	16:24	68	67.2	1	0	NR	NR	19	11
	Total	82	95	0.9174	33:62	N/A	N/A	1	0	N/A	N/A	150	106
UC	Moayyedi et al. [34]	38	37	18:20	02:11	42.2	35.8	3	2	NR	NR	NR	NR
	Rossen et al. [35]	23	25	11:12	11:14	40	41	0	0	18	16	21	24
	Paramsothy et al. [39]	41	40	22:19	01:15	35.6	35.4	2	1	32	33	78	80
	Costello et la. [41]	38	35	20:18	20:15	38.5	35	3	2	NR	NR	NR	NR
	Sood et al. [44]	31	30	22:09	22:08	33	34.6	0	0	NR	NR	24	20
	Total	171	167	93:78	104:63	N/A	N/A	8	5	50	49	123	124
Systemic sclerosis	Fretheim et al. [24]	5	4	00:05	00:04	58	66	0	1	5	4	35	13
Multidrug-resistant Enterobacteriaceae	Huttner ET AL. [42]	22	17	10:12	08:09	70	64	1	2	19	13	104	66
Obesity	Allegretti ET AL. [23]	11	11	01:10	01:10	44.5	43.2	0	0	NR	NR	26	25
	Yu et al. [28]	12	12	04:08	03:09	42.5	38.5	0	0	NR	NR	34	25
	Total	23	23	05:18	04:19	N/A	N/A	0	0	N/A	N/A	60	50
Alcohol Use Disorder	Bajaj et al. [37]	10	10	NR	NR	67.1	62.9	0	0	NR	NR	NR	NR
IBS	Halkjær et al. [12]	26	26	08:18	08:18	37.28	35.54	0	0	22	15	55	32
	Johnsen et al. [13]	55	28	12:19	19:09	44	45	0	0	2	3	2	3
	Aroniadis et al. [14]	48	48	06:18	06:18	37.31	37.31	0	0	NR	NR	23	24
	El-Salhy et al. [46]	109	55	1.0181	08:47	39.3	41.2	0	0	NR	NR	90	12
	Lahtinen et al. [47]	23	26	11:12	17:09	47.3	46.3	0	0	7	10	8	11
	Total	251	183	108:153	82:101	N/A	N/A	0	0	31	28	178	82
Constipation	Tia et al. [40]	30	30	11:19	09:21	53.1	55.4	0	0	NR	NR	50	4
Total		603	529	248:345	240:279	N/A	N/A	10	8	105	94	700	445

*IMT* intestinal microbiota transplantation, *CON* control, *M* male, *F* female, *NR* not reported, *SAEs* serious adverse events, *CAEs* common adverse events, *N/A* not applicable, *rCDI* recurrent Clostridioides difficile infection, *UC* Ulcerative colitis,* IBS* Inflammatory Bowel Disease

**Fig. 3:**
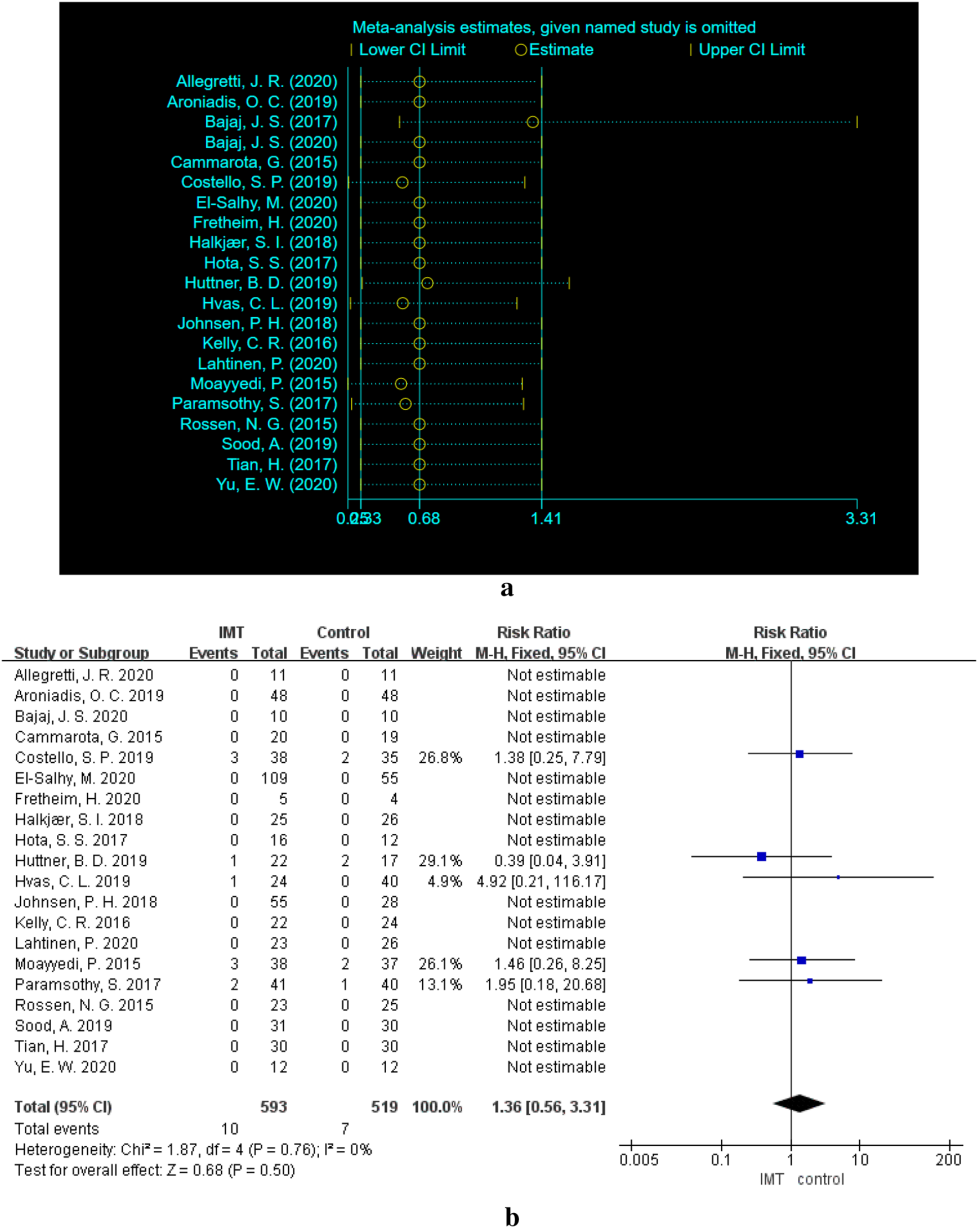
**a** Sensitivity analysis of the 21 studies related to SAEs. **b** SAEs of IMT group versus control group

The total number of patients with SAEs in the IMT group was 28, but only 10 SAEs were definitely/probably/possibly related to IMT. IMT-related SAEs occurred mainly in 5 studies [34, 39, 41–43] (Table 3). There were no deaths in the IMT arm. Meta-analysis of IMT-related SAEs in these 21 studies found that heterogeneity existed in the results. We conducted a sensitivity analysis of the 21 studies and found that one study (Bajaj [37]) had a great impact on heterogeneity (Fig. 3a), so it was removed considering that this study was the main source of heterogeneity. Finally, it was concluded that no significant difference was found in the incidence of SAEs between the IMT group and the control group. The pooled RR for the IMT group compared with the control group was 1.36 (95% CI 0.56–3.31, *P* = 0.50) and evidence showed that there was no heterogeneity between these 20 studies (*x*^2^ = 1.87, df = 4, *P* = 0.76, *I*^2^ = 0%) (Fig. 3b). Thus, the fixed effect model was adopted.

**Table 3:** Details of SAEs

	Moayyedi et al. [34]	Paramsothy et al. [37]	Costello et al. [41]	Huttner et al. [42]	Hvas et al. [43]
IMT	Two persons developed patchy inflammation of the colon and also rectal abscess formation, which resolved with antibiotic therapy One was Clostridium difficile toxin positive at the end of therapy	One patient with refractory ulcerative colitis who was assigned IMT withdrew at week 2 because of clinical and endoscopic deterioration and underwent colectomy One patient with moderately severe colitis who was assigned IMT remained unwell at week 3, withdrew from the study, and was admitted for intravenous corticosteroid therapy	One patient developed worsening colitis One patient developed clostridium difficile colitis requiring colectomy One patient developed pneumonia	Female, 57 years, Utrecht Patient with known liver cirrhosis and recurrent episodes of hepatic encephalopathy hospitalized 2 weeks after IMT for episode of encephalopathy	A 50-year-old woman developed a sepsis-like clinical picture with pyrexia, convulsions, vomiting, and diarrhea in her private home for 3 h the evening after an uncomplicated IMT delivered by colonoscopy. Although perceived life-threatening, the patient was not admitted to the hospital and had complete recovery within 24 h without further treatment
CON	One person in the placebo group developed worsening colitis and was admitted to hospital 3 weeks into the trial and had an urgent colectomy One person developed patchy inflammation of the colon and also rectal abscess formation, which resolved with antibiotic therapy	One patient with moderately severe colitis who was allocated placebo withdrew at week 3 and needed hospitalization	Two SAEs in the aIMT group (both worsening colitis)	Male, 68 years, Paris, Two hospitalizations for recurrent urinary tract infection Female 56 years, Utrecht, Hospitalization for pyelonephritis	No SAE

*IMT* intestinal microbiota transplantation, *aIMT* autoplastic intestinal microbiota transplantation, *CON* control, *SAE* serious adverse event, *SOC* standard of care, *HE* hepatic encephalopathy

**Fig. 4:**
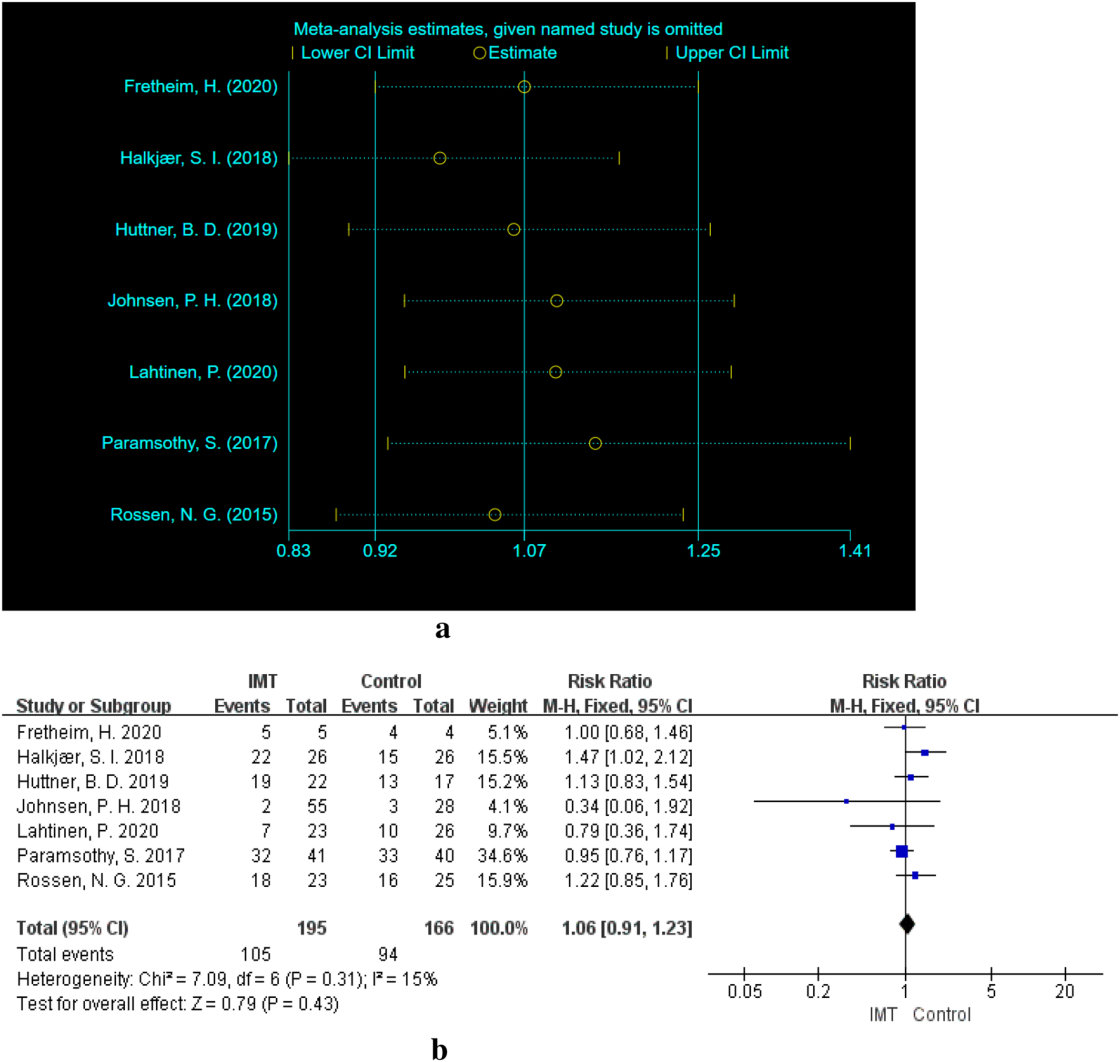
**a** Sensitivity analysis of the 7 studies related to CAEs. **b** CAEs of IMT group versus control group

Only 7 of the 20 studies reported the number of patients with CAEs [12, 13, 24, 35, 39, 42, 47], including 105 in the IMT group and 94 in the control group. There was no significant difference in the incidence of AEs between the IMT group and the control group (RR = 1.06, 95% CI  0.921–1.23, *P* = 0.43). There was no heterogeneity among the 7 articles (× ^2^ = 7.09, df = 6, *P* = 0.31, *I*^2^ = 15%) (Fig. 4a). Therefore, the fixed effect model was implemented. We also conducted a sensitivity analysis on the results and found no studies that had a large impact on heterogeneity (Fig. 1c).

### Different diseases and AEs of IMT

Of the RCT studies on IMT included in this analysis, namely, 4 studies on rCDI, 5 studies on ulcerative colitis (UC), 5 studies on IBS, and 2 studies on obesity, there was only one study on other related diseases, such as hepatic encephalopathy, systemic sclerosis, multidrug-resistant Enterobacteriaceae, alcohol use disorder, and constipation (Table 2).

As shown in Table 2, a total of 177 rCDI patients were included in the RCTs analysed in this study, 82 of whom received IMT treatment, and only one patient had an SAE with septic clinical manifestations, including fever, convulsions, vomiting, and diarrhoea. No SAEs were observed in rCDI patients in the control group. In the four RCT studies on rCDI, IMT patients were most likely to experience AEs, including diarrhoea, abdominal pain, abdominal distention, nausea, vomiting, and constipation, but most of the AEs lasted for a short time and resolved spontaneously. Hota et al. found that abdominal pain and abdominal distention were equally common in the IMT and vancomycin groups, but abdominal pain, abdominal distention, mucoid stools, and foul-smelling stools were more common in the vancomycin group at a later stage [38]. In the RCT study by Kelly et al., it was found that autologous IMT was more prone to cause chills than donor IMT, while there was no significant difference in the incidence of other AEs [36]. After 8 weeks of follow-up after IMT treatment, Hvas et al. found that one patient had small bowel bacterial overgrowth after primary IMT and that there were no statistically significant changes in patients' body weight, plasma albumin, or haemoglobin [43]. Most RCT studies of IMT for rCDI did not find any recurrence or death during follow-up.

**Fig. 5:**
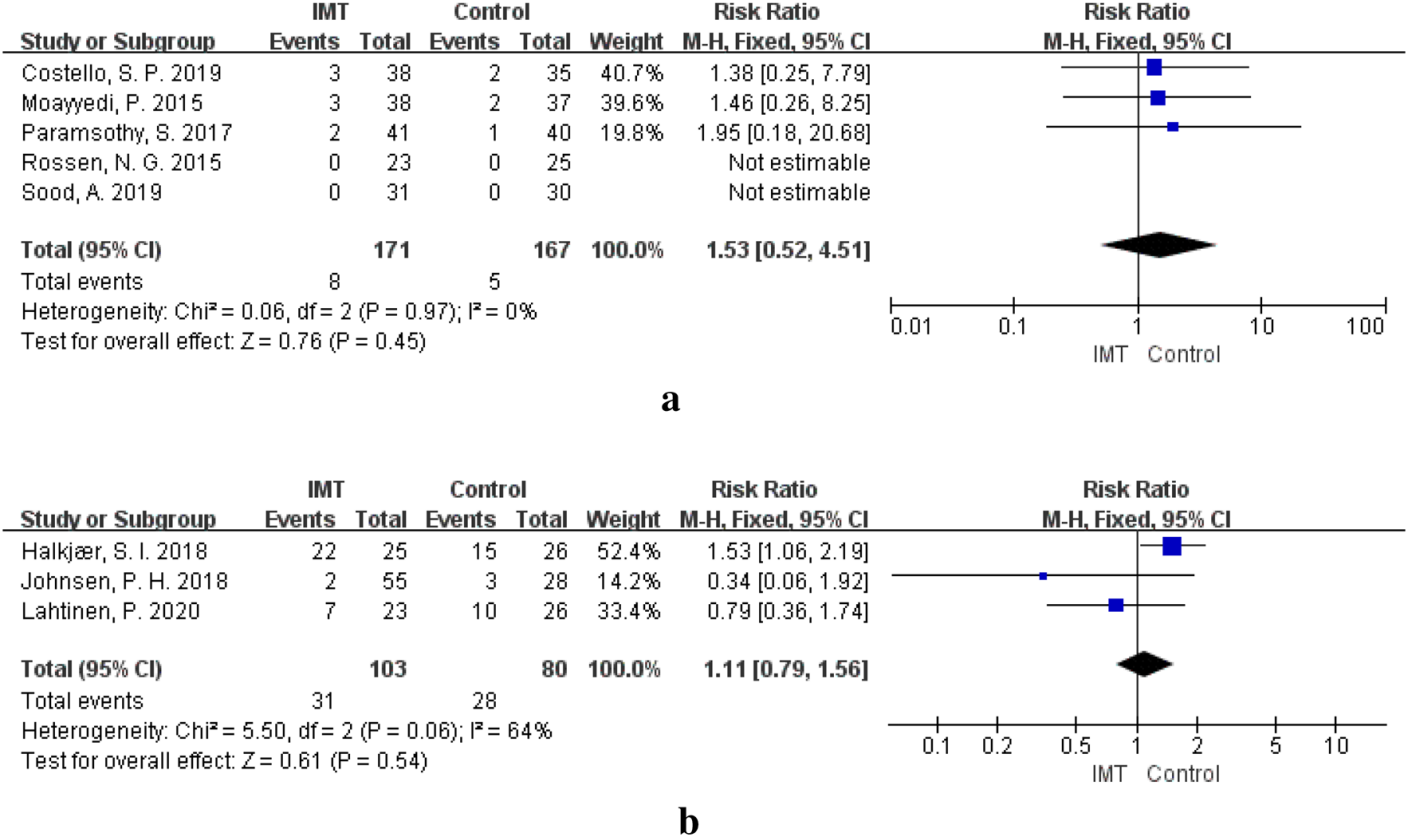
**a** SAEs of IMT in patients with UC. **b** CAEs of IMT in patients with IBS

Of the enrolled UC patients, 171 received IMT, and 8 developed IMT-related SAEs, such as varying degrees of infection and disease progression. However, there was no significant difference in the incidence of SAEs between the IMT group and the control group (RR = 1.53, 95% CI 0.52–4.51, P = 0.45) (Fig. 5a). No individual donor or donor batch was significantly associated with the primary outcome or SAEs, although the study was not powered to evaluate this possibility [39]. Although other SAEs, such as intestinal perforation and cytomegalovirus infection, were found during the follow-up of these studies, they are not considered to be related to IMT [35]. Similarly, data collected in this study showed no significant difference in the number of CAEs associated with IMT treatment of UC. Paramsothy et al. showed that 32 of 41 IMT patients (78%) and 33 of 40 placebo patients (83%) had at least one AE during 8 weeks of IMT treatment. There was no significant difference in the number or type of AEs between the two groups. The most common AE was self-limiting gastrointestinal disorders [39]. In addition, Costello et al. found that three participants developed new anaemia (control, 2; IMT, 1), 2 had a mild elevation in alkaline phosphatase (control, 0; IMT, 2), and 4 had mild elevations of alanine aminotransferase (control, 3; IMT, 1). However, there were no significant differences between the IMT and control groups [41].

Huttner et al. conducted a study to evaluate whether oral antibiotics followed by IMT can eradicate intestinal carriage with the β-lactamase Enterobacteriaceae and carbapenemase-producing Enterobacteriaceae. Among the 21 patients in the IMT group, 19 (90%) experienced at least one CAE (overall, 104 CAEs). There were four SAEs, of which only one was possibly related to IMT (a patient with known liver cirrhosis and recurrent episodes of hepatic encephalopathy hospitalized two weeks after FMT for an episode of encephalopathy) [42].

In the included IBS studies, there were no SAEs in either the IMT group or the control group [12–14, 46, 47]. There was no significant difference in the incidence of CAEs in IBS studies (RR = 1.11, 95% CI 0.79–1.56, P = 0.54) (Fig. 5b). CAEs mainly include diarrhoea, abdominal pain, abdominal distention, constipation, and other gastrointestinal symptoms, among which the most common AE is diarrhoea [12], but most of these cases are self-limited. Some patients may experience transient fever. Dizziness and nausea in some patients may be related to the medication and instrumentation used during colonoscopy [13]. No IMT-related AEs were observed during follow-up after IMT treatment. In a study of IMT for slow-transit constipation included in this review, no SAEs were found. The CAEs reported were mainly in the IMT group (IMT, 50; control, 4), including exhaust, nausea, abdominal pain, and diarrhoea, which were transient. Since IMT was administered through the nasointestinal tube in this study, some AEs, such as nausea and dyspnoea, were considered to be related to the IMT delivery method [40].No SAEs were found in the IMT group in the RCT studies of IMT treatment for extraintestinal diseases such as hepatic encephalopathy, systemic sclerosis, alcohol use disorder, and obesity. In the IMT group of the hepatic encephalopathy study, one patient at day 84 post-IMT with acute kidney injury responded within 24 h to intravenous hydration and one at day 115 due to chest pain that was ruled out for an acute cardiac event. However, both cases were judged to be unrelated to IMT [37]. In the systemic sclerosis study, patients in the FMT group reported more postinterventional CAEs than the placebo controls, but all the CAEs were regarded as mild and transient, including abdominal bloating, diarrhoea, nausea, and constipation. There was one duodenal perforation in the control group that was related to endoscopy, not placebo [24]. In the obesity studies, most of the CAEs in the IMT group and the control group were mild abdominal pain and diarrhoea, with no significant difference between the two groups [23, 48].

### Fresh or frozen faecal sample and AEs

**Fig. 6:**
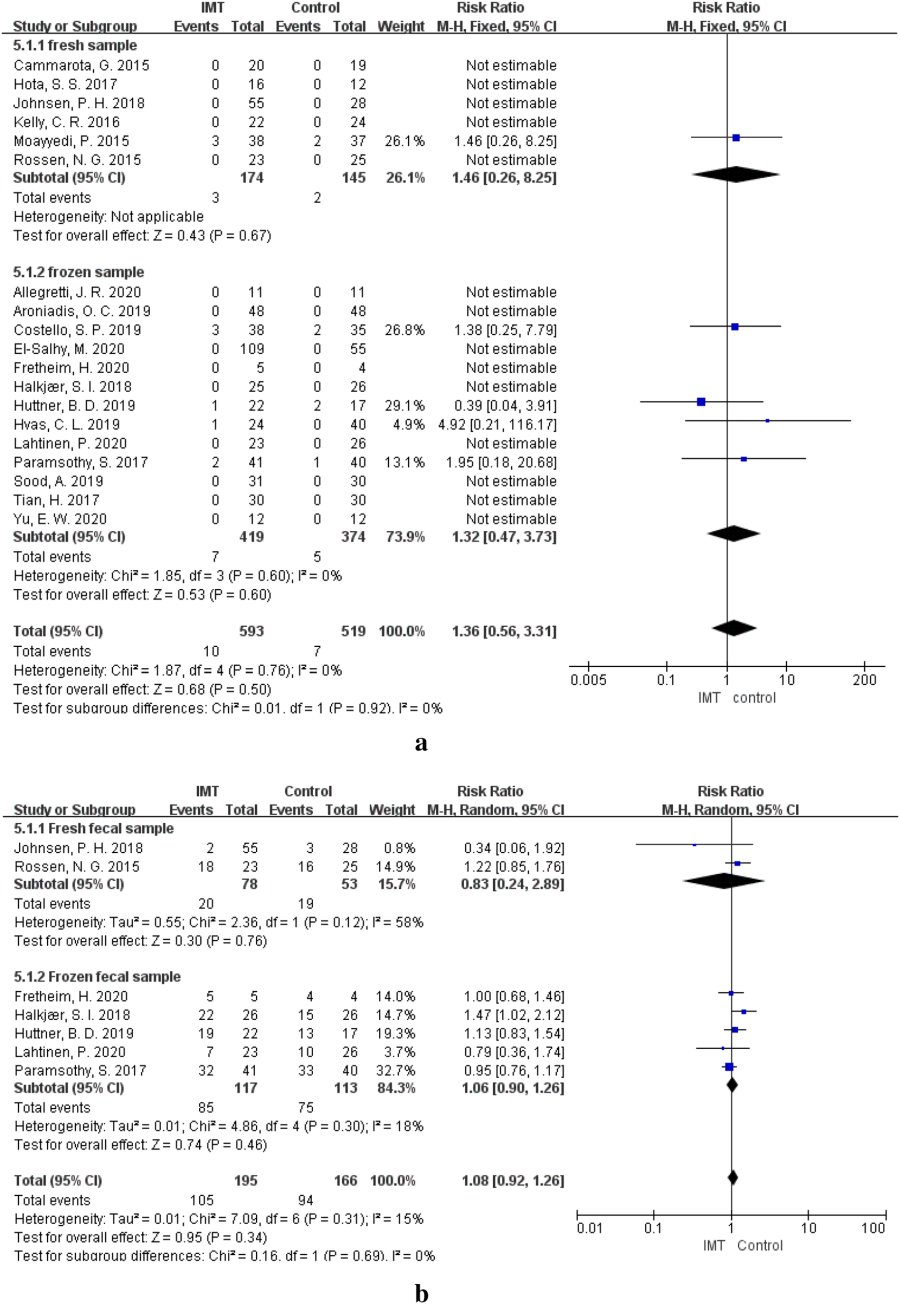
**a** SAEs of different types of fecal sample. **b** CAEs of different types of fecal sample

Six [13, 33–36, 38] of the 20 studies used fresh faecal samples, and 13 [12, 14, 23, 24, 39–48] used frozen stool samples for IMT. The occurrence of SAEs (RR = 1.46, 95% CI  0.26–8.25, *P* = 0.67) and CAEs (RR = 0.83, 95% CI 0.24–2.89, *P* = 0.76) was not different between the IMT group and the control group when IMT group patients used fresh faecal samples. When frozen faecal samples were used for IMT, no difference was observed between the IMT group and the control group in the incidence of SAEs (RR = 1.32, 95% CI  0.47–3.73, *P* = 0.60) and CAEs (RR = 1.06, 95% CI  0.90–1.26, *P* = 0.46). The incidence of SAEs and CAEs was not different between faecal sample type subgroups (*P*_(SAE)_ = 0.92, *P*_(CAE)_ = 0.69). (Fig. 6, Table 4).

**Table 4:** AEs of IMT by subgroups

subgroup	SAEs				CAEs			
	Number of studies	Event rate of IMT (%)	RR, 95% CI, over effect P-value	Subgroup differences	Number of studies	Event rate of IMT (%)	RR, 95%CI, over effect P-value	Subgroup differences
				P-value				P-value
Type of stool sample				0.92				0.69
Fresh	6	1.72	RR = 1.46, 95%CI 0.26–8.25, P = 0.67		2	25.64	RR = 0.83, 95% CI 0.24–2.89, P = 0.76	
Frozen	13	1.67	RR = 1.32, 95% CI 0.47–3.73, P = 0.60		5	72.65	RR = 1.06, 95% CI 0.90–1.26, P = 0.46	
Delivery route				0.25				0.04
UGI	6	0.50	RR = 0.34, 95% CI 0.06–2.10, P = 0.25		3	84.00	RR = 1.15, 95% CI 0.93–1.43, P = 0.20	
LGI	11	2.72	RR = 1.53, 95% CI 0.21–3.53, P = 0.45		3	34.45	RR = 0.86, 95% CI 0.68–1.10, P = 0.23	
Both UGI and LGI	1	4.17	RR = 4.92, 95%CI 0.21–116.17, P = 0.32		0	–	–	
Capsules	3	0	–		1	84.62	RR = 1.47, 95% CI 1.02–2.12, P = 0.04	
Frequency				0.4				0.32
More than once	12	2.65	RR = 1.18, 95% CI 0.46–3.04, P = 0.74		4	81.25	RR = 1.13, 95% CI 0.93–1.37, P = 0.21	
once	8	0.38	RR = 4.92, 95% CI 0.21–116.17, P = 0.32		3	16.87	RR = 0.85, 95% CI 0.49–1.45, P = 0.55	
Dosage				0.48				0.86
Greater than 50 g	9	1.80	RR = 1.72, 95% CI 0.56–5.23, P = 0.34		2	29.63	RR = 0.88, 95% CI 0.20–3.80, P = 0.87	
Less than 50 g	6	1.24	RR = 0.87, 95% CI 0.19–4.01, P = 0.86		2	80.95	RR = 1.0, 95% CI 0.84–1.20, P = 0.98	

*IMT* intestinal microbiota transplantation, *SAEs* serious adverse events, *CAEs* common adverse events, *RR* relative risk, *CI* confidence interval

### IMT delivery methods and AEs

**Fig. 7:**
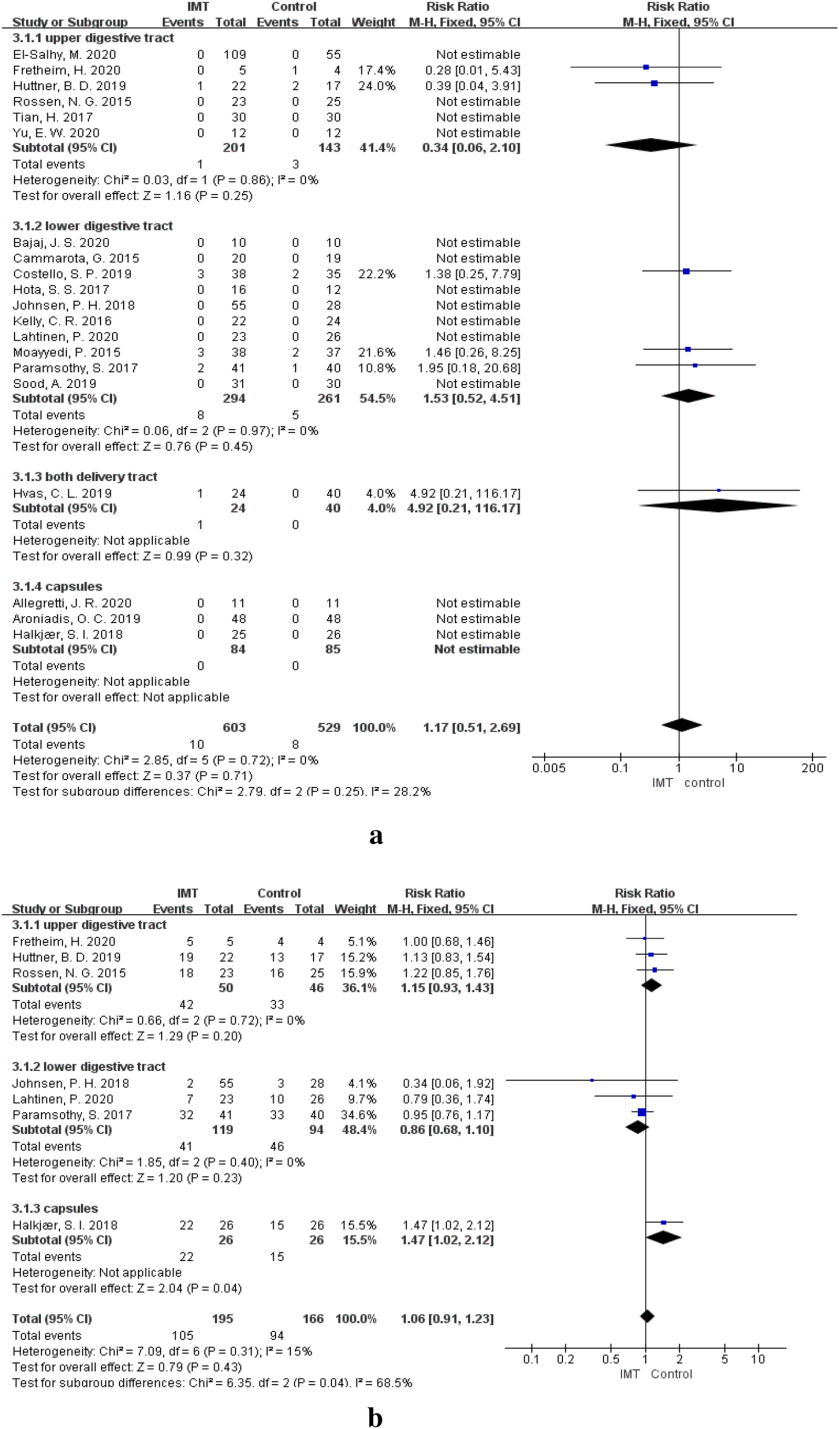
**a** SAEs of IMT in different delivery methods. **b** CAEs of IMT in different delivery methods

To observe the relations between delivery methods and AEs, the delivery methods in these studies were divided into upper gastrointestinal (UGI) delivery (via a nasoduodenal tube, nasojejunal tube, or gastroscope) [24, 35, 40, 42, 46, 48], lower gastrointestinal (LGI) delivery (via an enema or colonoscopy) [13, 33, 34, 36–39, 41, 44, 45, 47], both UGI and LGI delivery [43], and oral capsules [12, 14, 23]. When patients were treated with IMT delivery LI routes, no significant difference was observed between the IMT group and the control group in the incidence of SAEs (RR = 0.34, 95% CI 0.06–2.10, *P* = 0.25) and CAEs (RR = 1.15, 95% CI 0.93–1.43, *P* = 0.20). In terms of LGI delivery, the incidence of SAEs (RR = 1.53, 95% CI 0.52–4.51, *P* = 0.45) and CAEs (RR = 0.86, 95% CI 0.68–1.10, *P* = 0.23) was also not significantly different between the two groups. One study used delivery methods via colonoscopy or nasojejunal tubes, and the incidence of SAEs was not significantly different between the IMT group and the control group (RR = 4.92, 95% CI 0.21–116.17, *P* = 0.32). No SAEs were observed in patients who underwent IMT and placebo treatment with oral capsules, but the incidence of CAEs in the IMT group was significantly higher than that in the control group when patients were treated with IMT via oral capsules (RR = 1.47, 95% CI 1.02–2.12, *P* = 0.04). No difference was observed in the incidence of SAEs between subgroups of delivery (*P*_(SAE)_ = 0.25). However, the incidence of CAEs was significantly different between subgroups of delivery methods (*P*_(CAE)_ = 0.04). (Fig. 7, Table 4).

### Frequency and dosage of IMT and AEs

**Fig. 8:**
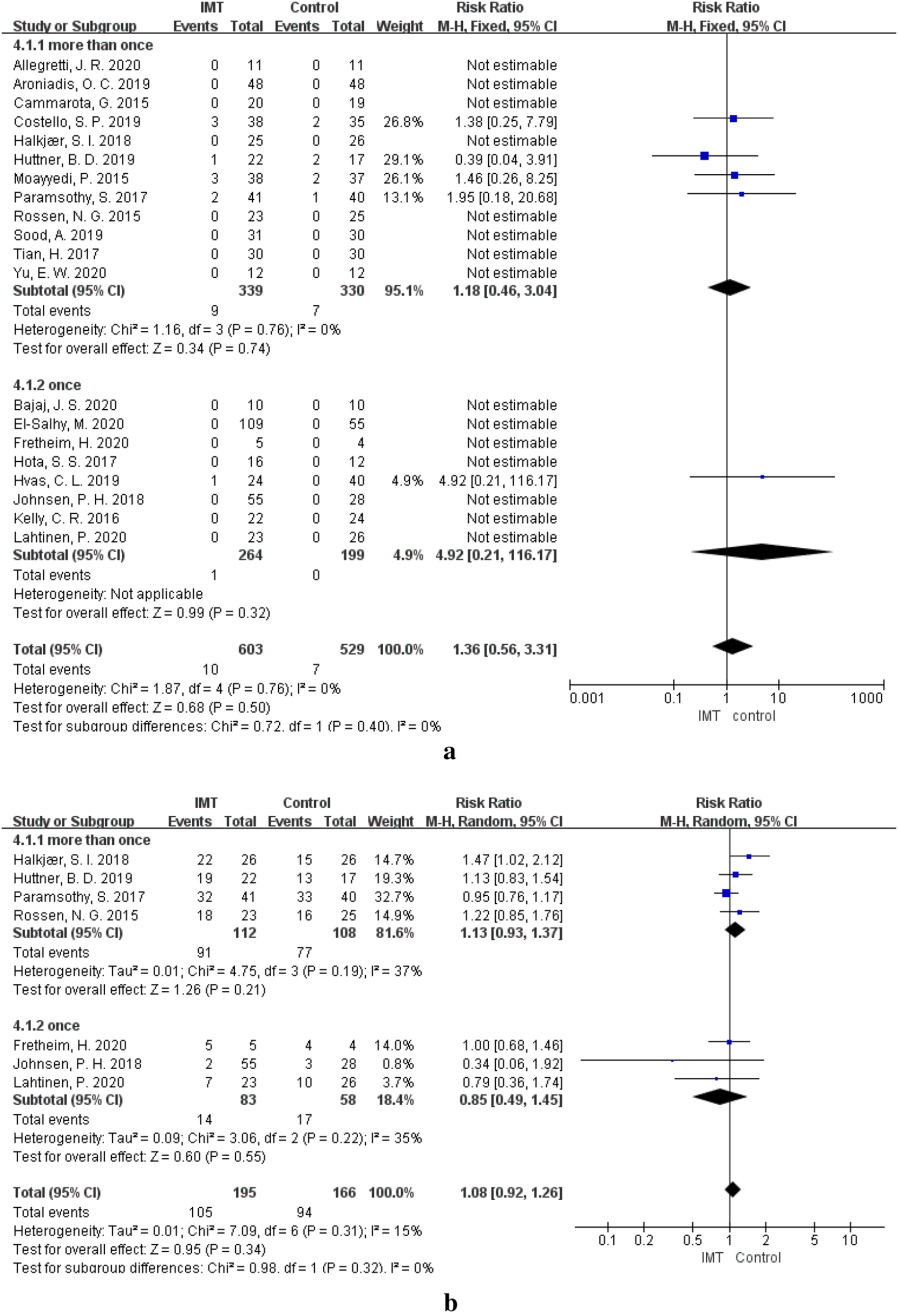
**a** SAEs of different IMT frequencies. **b** CAEs of different IMT frequencies

**Fig. 9:**
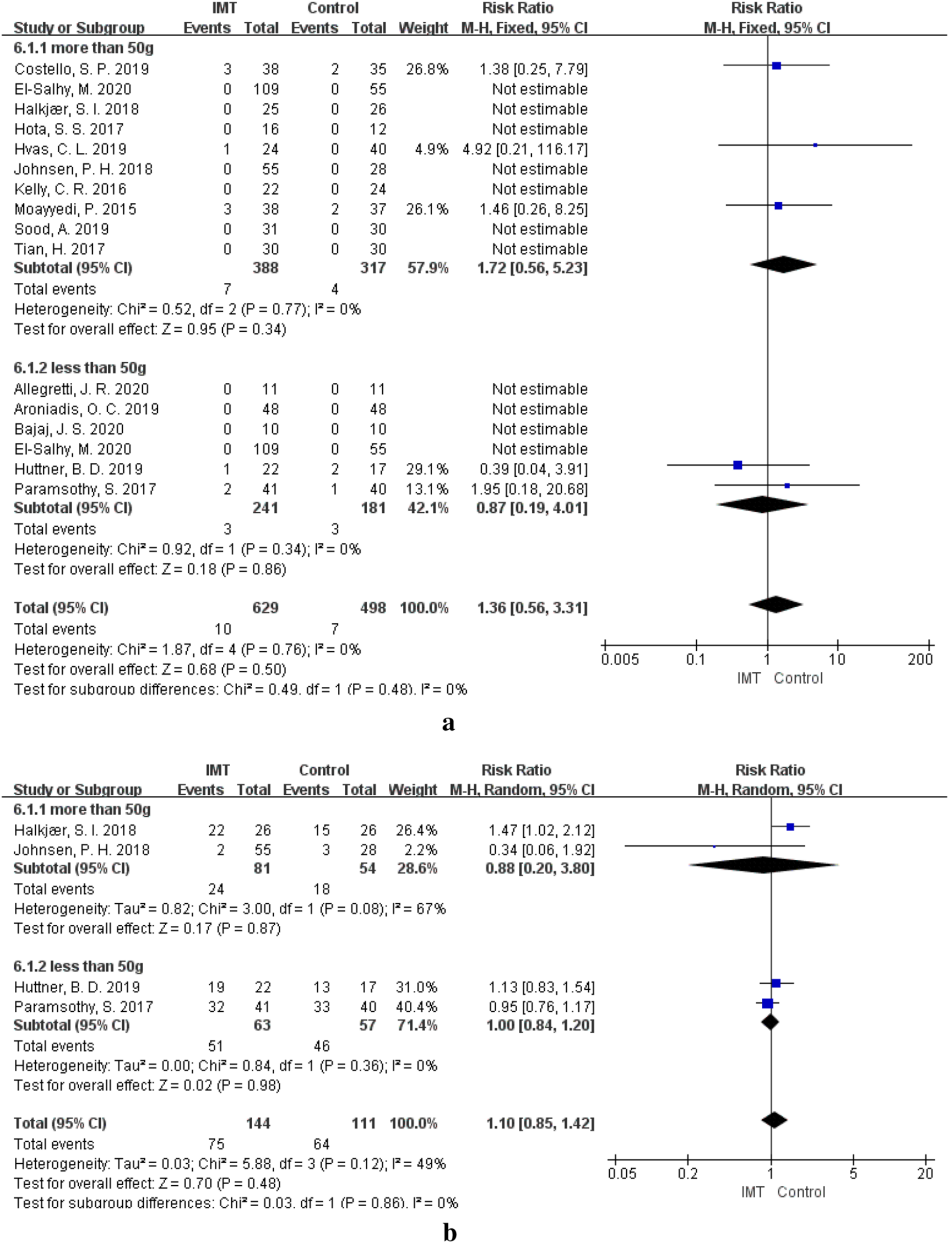
**a** SAEs of different IMT dosage. **b** CAEs of different IMT dosage

The incidence of AEs at different frequencies of IMT was observed. Compared to that in the control group, there was no difference in the incidence of SAEs (RR = 1.18, 95% CI 0.46–3.04, *P* = 0.74) or CAEs (RR = 1.13, 95% CI 0.93–1.37, *P* = 0.21) in the patients treated with IMT more than once [12, 23, 33–35, 39–42, 44, 48]. When patients were treated with IMT only once [13, 14, 24, 36, 38, 43, 45–47], there was also no significant difference in the incidence of SAEs (RR = 4.92, 95% CI 0.21–116.17, *P* = 0.32) and CAEs (RR = 0.85, 95% CI 0.49–1.45, *P* = 0.55) compared with the corresponding values in the control group (Fig. 8, Table 4). The dosage of stool samples used for IMT ranged from 37.5 g to 100 g in these studies. When the IMT dosage was greater than 50 g [13, 34, 36, 38, 40, 41, 43, 44, 46], there was no obvious difference compared with the control group in the incidence of SAEs (RR = 1.72, 95% CI 0.56–5.23, *P* = 0.34) and CAEs (RR = 0.88, 95% CI 0.20–3.80, *P* = 0.87). The incidence of SAEs (RR = 0.87, 95% CI 0.19–4.01, *P* = 0.86) and CAEs (RR = 1.0, 95% CI 0.84–1.20, *P* = 0.98) was also not different between the IMT group and the control group when the IMT dosage was less than 50 g [14, 23, 39, 42, 45, 46]. In both the frequency subgroups (*P*_(SAE)_ = 0.40, *P*_(CAE)_ = 0.32) and the dosage subgroups (*P*_(SAE)_ = 0.48, *P*_(CAE)_ = 0.86), no difference was observed in the incidence of SAEs and CAEs (Fig. 9, Table 4).

### Publication bias

**Fig. 10:**
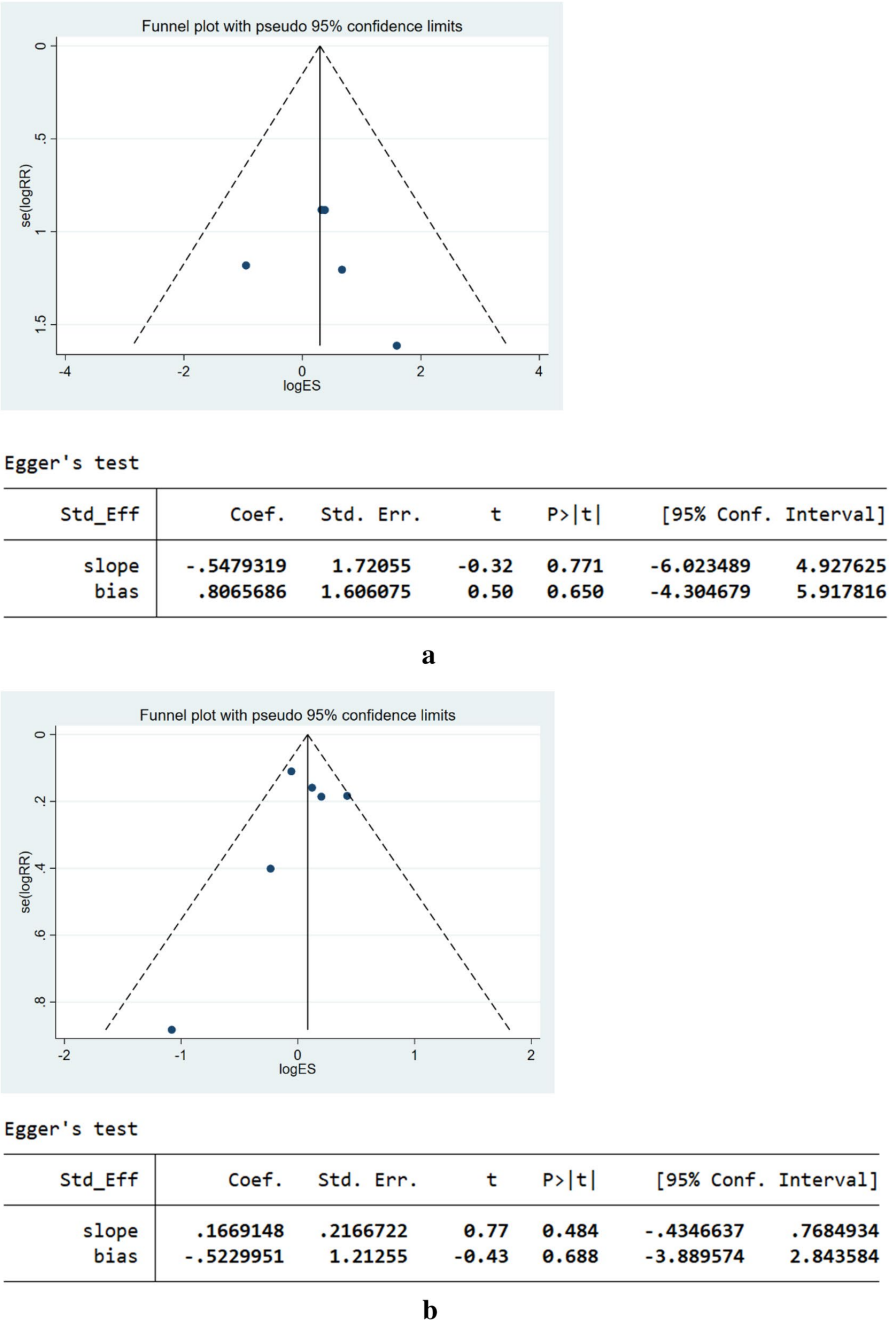
**a** Funnel plot and Egger’s test of SAEs. **b** Funnel plot and Egger’s test of CAEs

The funnel plot did not indicate potential publication bias in terms of SAEs and CAEs (Fig. 10), both of which were confirmed by Egger’s test (*P*_(SAE)_ = 0.650; *P *_(CAE)_ = 0.688).

## Discussion

Although the current expert consensus recognizes that the specific indication for IMT is rCDI [49], its promising efficacy has been gradually demonstrated in other diseases [50, 51]. Some studies have only focused on its efficacy while ignoring its AEs. With the wide application of IMT, it is necessary to comprehensively evaluate its safety so that clinicians and patients can make a choice by balancing the advantages and disadvantages and promoting the standardization of IMT.

This systematic review synthesized evidence from 20 RCTs fulfilling the inclusion criteria that were identified and eligible and evaluated the incidence of SAEs related to IMT. One study on hepatic encephalopathy was not included in the meta-analysis of SAEs due to its large impact on heterogeneity. Considering that the control treatment in this study, unlike most of the other included studies, was not placebo or autologous IMT but standard care alone, it is also possible that its pathophysiology is inconsistent with other diseases. When data from all studies were pooled, there was no significant difference between the IMT group and the control group in the incidence of SAEs. Of the 20 studies, 16 described IMT-related CAEs, but only 7 described the number of patients with CAEs. These 7 studies were included in a meta-analysis of the incidence of CAEs. The results showed no significant difference in the incidence of CAEs between the IMT and control groups. However, the incidence of CAEs was significantly different between different delivery routes, but there was no significant difference in other specific subgroups. It is believed that the risk factors for IMT-related AEs in patients mainly include low immunity of the recipient [49], intestinal mucosal barrier injury [31], unqualified faecal sources [52], contamination in the faecal bacteria production process, and IMT delivery methods. Except for those due to the IMT delivery methods [30], AEs due to the other four risk factors were considered to be related to the application of faecal bacteria.

Currently, IMT is widely used in the treatment of diseases such as rCDI, IBD, IBS, autoimmune diseases, metabolic disorders, and mental disorders. Although the efficacy of IMT in the treatment of these diseases has been widely reported and affirmed, more attention should be given to the occurrence of AEs. Interestingly, the patients with IMT-related SAEs in the studies included in this systematic review were all individuals with compromised immunity and impaired intestinal mucosal barriers (including conditions such as UC, rCDI, and multidrug-resistant bacterial infections). However, no SAEs associated with IMT have been observed in studies of IBS, constipation, or other extraintestinal diseases without intestinal mucosal damage. A systematic review published by Zhang et al. also showed that all IMT-related SAEs occurred in patients with mucosal barrier injury. They noted that 8 of these patients developed bacteraemia, of which 5 cases were associated with IMT: one patient died of severe sepsis, three patients tested positive for drug-resistant *Escherichia coli*, and one developed klebsiellosis shortly after colonoscopy IMT [31]. Angelberger et al. observed that most AEs may be caused by bacteria entering the intestinal tract [53]. Therefore, we believe that patients with compromised immunity and an impaired intestinal mucosa barrier may be more prone to fever, diarrhoea, constipation, allergy, and other AEs related to infection and immunity. Interestingly, in a study using IMT to treat immunocompromised patients with CDI, there were no cases of infection significantly associated with IMT, but the researchers suggested that IMT is not without risk, which may be greater in acutely ill patients. We agree with this point of view. As seen from the UC-related studies we included, IMT-related SAEs occurred only in patients with active UC [34, 39, 41]. However, there were no associated SAEs when IMT was administered to UC patients with UC in clinical remission [44]. In the experience of several of the coauthors, there is limited efficacy to a single application of IMT performed at the time of acute CDI, and delaying IMT or performing a second IMT after the patient has finished a course of anti-CDI therapy may be the best course [54]. For patients with severe immunosuppression and severe intestinal mucosal injury, the consensus of Chinese experts includes them in the exclusion criteria of IMT treatment [55]. Therefore, the use of IMT should be more cautious for patients with low immunity and intestinal mucosa injury. If possible, IMT is recommended to be avoided in the acute phase of the disease in such patients or to be used as an adjunct or consolidation therapy.

Ensuring that faecal sources are qualified and avoiding contamination during the production and infusion of faecal bacteria are also important measures to prevent IMT-related AEs in patients [3, 56]. To improve safety, many studies recommend using faecal donations by parents, spouses, relatives and friends of children in the same environment as the patient [57, 58]. However, according to current studies, it has not been found that the efficacy and safety of the faecal bacteria provided by the donor of patient choice is higher than that provided by other healthy volunteers [59]. Since most of the donors included in this review were healthy volunteers, it was not possible to further analyse the differences between the two types of donors. Although the donors had been tested for viruses, intestinal pathogens, parasites and ova before donation, donors who were in the latent period of an infection could not be excluded by the above screening tests; thus, these donors might have contributed to the development of infectious AEs. For example, two studies reported viral infections (cytomegalovirus and norovirus). Cytomegalovirus infection occurred after home IMT, which was suspected to be related to a child donor without strict donor screening [60]. Norovirus infection was speculated to be probably related to environmental pollution by an endoscopy suite employee [61]. One patient who received IMT via a capsule developed drug-resistant *Escherichia coli* bacteraemia that was assumed to be transmitted by IMT, and the patient died of severe sepsis 10 days post-IMT [62]. Therefore, a consensus report from a multidisciplinary UEG working group pointed out that IMT products should be put under quarantine until the donor has been found acceptable in a repeat screen. For severely immunocompromised patients, the prepared IMT preparation itself should undergo quality control that includes (re)screening for potential pathogens [49]. However, unidentified pathogens carried by the donor may induce AEs [63]. It has been reported that among the 8 cases of infections possibly related to IMT, 4 cases involved infection by unknown pathogens. In addition, individual donor differences may also lead to AEs, such as a patient who developed fever, vomiting and tachycardia after receiving faecal transplantation from her brother, while her niece’s faecal transplantation was well tolerated [64]. Cytomegalovirus infection, which is rare in IMT reception, may be caused by a young donor [60]. There is also a do-it-yourself (DIY) approach to IMT, which is used more frequently in IBD and IBS patients, with 12% of patients reporting AEs such as abdominal pain, flatulence, mood changes, fever, infection and hospitalization [65]. These may be caused by nonstandardized household or self-prepared IMT methods. Therefore, it is necessary to raise awareness of DIY-IMT and reduce the incidence of AEs. To improve the safety of IMT, washing microbiota transplantation (WMT) has been continuously researched in China. A study showed that more types and quantities of viruses and proinflammatory agents are removed during the cleaning process, enhancing the safety of WMT [66]. The IMT standardization study group released the WMT methodology consensus in 2020 based on evidence that washed microbiota preparation could reduce IMT-related AEs [66–68]. Although studies have reported significant differences between faecal types in the assessment of AEs, they also indicated a higher incidence of frozen faecal materials. However, this result should be interpreted with caution, as most of the included studies did not standardize AE reporting, which could be biased [69]. In our study, no difference was found in the incidence of AEs between the use of fresh and frozen faecal materials.

A large number of studies have shown that the delivery route is an important factor in the occurrence of AEs in IMT [52]. IMT via the UGI route is prone to cause nausea, vomiting, nasal congestion, sore throat, abdominal pain, abdominal distension, aspiration, asphyxia and other adverse reactions [70–72], while IMT via LGI delivery is prone to result in abdominal pain, increased stool frequency, abdominal distension, cramps, anorectal discomfort, rectal abscess and other conditions [30, 34]. It is generally believed that transplantation via UGI delivery is more prone to AEs [73]. A significant difference was observed in the incidence of CAEs between different IMT delivery routes in our study. Although the CAEs related to IMT delivery are not very harmful, they can cause severe discomfort and lead to termination of IMT [74], especially IMT performed via the UGI tract. Therefore, while considering the efficacy of IMT treatment, the use of IMT methods that minimize patient discomfort, such as oral faecal capsules or colonic transendoscopic enteral tubing (TET), should be considered. One study showed that oral frozen capsule IMT relieved diarrhoea in patients with recurrent episodes of CDI, and they rarely experienced AEs such as abdominal cramps and bloating [75]. In our opinion, AEs caused by the delivery routes were actually avoidable, as most of these were probably due to operational errors or improper handling. To avoid the occurrence of such SAEs, we should try to avoid or reduce invasive IMT methods, and the operation should be gentle and cautious. We should closely observe the patient's vital signs and reactions.

Our study found that the probability of multiple IMTs yielding AEs was significantly higher than that of single IMTs, although the difference was not statistically significant. However, if repeated IMT is needed, it is recommended to use an indwelling catheter of the digestive tract or an oral faecal bacteria capsule. In addition, although our study did not find a significant increase in the incidence of AEs during the use of high-dose faecal bacteria for IMT, for patients with poor digestive tract dynamics, excessive bacterial fluid infusion at one time is highly likely to aggravate nausea, vomiting, abdominal distension, abdominal pain and other AEs and may even lead to aspiration and asphyxia [76]. Therefore, the gastrointestinal motility of patients should be fully evaluated before IMT, especially IMT via the UGI route. If there is a gastrointestinal motility deficiency, the infusion dose should be reduced, the infusion speed should be slowed down, or drugs should be used to improve gastrointestinal motility. Bota Cui et al. noted that fewer AEs occurred in patients who received metoclopramide prior to IMT, suggesting that metoclopramide may help avoid AEs [77]. We believe that the routes of administration of IMT should be individualized based on patient and disease characteristics while considering the risks of potential AEs and the availability of different IMT products [52].

In addition to the AEs described above, it is not clear whether IMT recipients are at increased risk for certain diseases. These diseases are thought to have a causal relationship with the microbiome but have not been seen in the donor. For example, gut microbes that contain pathogens of certain diseases, such as *Fusobacterium nucleatum*, carry a risk of colon cancer [78]. Thus, one can ask whether this predisposition might be transferable to a recipient from a donor who is still healthy but will go on to develop colorectal carcinoma some years later. Even if these considerations are theoretically valid, there is no such evidence. In one set of cases, 31 patients with rCDI received a microbiome from a healthy donor. Two months later, the donor developed bloody diarrhoea and was found to have Crohn’s disease with ileocecal involvement. Notably, none of the IMT recipients developed any type of IBD during further follow-up, but this did not preclude the possibility that the observation time was too short [79]. Based on a single case report from the United States, it was long presumed that IMT could affect the body weight of the recipient [80]. Brant et al. described new autoimmune or rheumatic diseases that may occur after IMT. It is not clear whether these new diseases are related to IMT [81]. Therefore, current donor screening protocols and the long-term safety of IMT need to be questioned and studied.

The safety of IMT is one of the greatest concerns related to its use, but the AEs of IMT are generally underreported [30, 82]. Therefore, a strict supervision mechanism should be established for donor screening as well as faecal collection, preparation, preservation, distribution and later clinical application to ensure the standards and safety of all links. AEs should be strictly observed during and after IMT, and strict follow-up should be conducted [83]. IMT is different from other treatments in that it was rapidly implemented for widespread clinical use, bypassing the drug-development procedure, which typically collects prospective efficacy and safety data on large numbers of patients before making a treatment available [84, 85]. To assess the long-term safety, it is important to collect real-world evidence for the safety of the procedure. In the USA and Europe, IMT recipient registries have been launched to track efficacy and safety outcomes in adult and paediatric patients after IMT [86–88].

## Limitations

Current systematic reviews and meta-analyses have some limitations. Most of these studies did not describe the CAEs of IMT in detail. Most CAEs generally overlapped, and some patients experienced more than one AE, but the authors did not elaborate on this aspect, leading to the inability to analyse the incidence of CAEs in some studies. In addition, the lack of data related to the IMT procedure in some studies results in an inability to estimate the relationship between IMT procedure characteristics and AEs. In the studies included in this review, the donors were all healthy volunteers, so it was not possible to compare the incidence of AEs from different sources of faecal bacteria. However, all the included studies were RCTs with high quality and relatively reliable data, especially the detailed description of SAEs. Therefore, the correlation analysis of SAEs was comparatively reliable. No significant publication bias was found in the included papers.

## Conclusion

Although IMT is currently used in a variety of diseases with obvious efficacy, its associated AEs should not be ignored. For patients with low immunity and intestinal mucosal barrier injury, their condition should be fully evaluated before IMT, and donor faecal bacteria should be strictly screened to avoid contamination of the faecal bacteria production process and thereby prevent the occurrence of AEs. In addition, the IMT pathway is one of the high-risk factors for AEs; consequently, efficient and low-risk transplantation methods should be selected as far as possible during IMT, and invasive transplantation methods should be minimized or avoided. Furthermore, IMT-related operations should be performed carefully, gently and cautiously to avoid patient SAEs caused by operational errors. Currently, the long-term AEs of IMT are not clear, and long-term follow-up should be conducted [49, 55]. Therefore, to improve the safety of IMT, a strict quality control mechanism should be established for all steps of IMT, and emergency plans for AEs should be formulated to reduce the occurrence of AEs and to deal with AEs in a timely manner when they occur.

## Data Availability

Data are available upon request. Please contact the author for data requests.

## Abbreviations

IMT: Intestinal microbiota transplantation
rCDI: Recurrent *Clostridium difficile* infection
RCT: Randomized controlled trials
AE: Adverse event
SAE: Serious adverse event
CAE: Common adverse event
IBS: Irritable bowel syndrome
IBD: Inflammatory bowel disease
PRISMA: Preferred Reporting Items for Systematic Reviews and Meta-analyses
MeSH: Medical subject headings
CTCAE: Common Terminology Criteria for Adverse Events
PPI: Proton pump inhibitor
UC: Ulcerative colitis
UGI: Upper gastrointestinal
LGI: Lower gastrointestinal
WMT: Washing microbiota transplantation
TET: Transendoscopic enteral tubing
